# Evaluation of hooves’ morphometric parameters in different hoof trimming times in dairy cows

**Published:** 2013

**Authors:** Ahmadreza Mohamadnia, Amin Khaghani

**Affiliations:** 1 *Department of Clinical Sciences, Faculty of Veterinary Medicine, Ferdowsi University of Mashhad, Mashhad, Iran; *; 2 *Behboud Dam Sepahan Co., Esfahan, Iran.*

**Keywords:** Cow, Hoof trimming, Hoof, Morphometry

## Abstract

Wide variety of timings and techniques has been used based on the housing, production, availability of requirements etc. This current study was done for a morphologic evaluation of the cow's digit in different trimming times. A dairy herd with 2200 milking cow, free stalls barns, average daily production of 36 liters per cow was selected. Forty cows were assigned to four groups based on hoof trimming times as, 100-120 days in milk (Group I), cows before drying (Group II), visual long toed cows (Group III) and delayed pregnant cows (Group IV). Toe length from coronary band to the toe tip (A), dorsal hoof angle (D), toe height from coronary band in toe region to the ground level (B), heel height from coronary band in heel region to the ground level (C) and heel height to toe length proportion was measured. The highest toe length was recorded in medial digit of group IV (9.19 ± 0.68 cm) and the lowest one recorded in lateral digit of group I (8.28 ± 0.62 cm). Distribution of the cows in different groups under study was based on their toe length, as the highest and lowest distribution were recorded in groups III and I, respectively. The highest measurements in all indices were recorded in group IV that followed by group III except for toe height that was higher in group II following group IV. The lowest toe length was recorded in group I that needs more attention due to the risk of over trimming and its supposed complications in this group.

## Introduction

Lameness is a result of changes in locomotion system of the cow that may be produced by congenital abnormalities, infectious, non-infectious, metabolic conditions and trauma,^1^ and is placed in the third place after infertility and reproduction disorders in health issues of the cows. Lameness may result in less milk production, however, it is the most important economic loss resulted from infertility and early culling of the affected animals.^2^ High milk production is a risk factor for lameness and lameness can decrease milk production.^3^ However, Haskel *et al*. did not find any correlation between high milk production and increase risk of lameness.^4^ Concrete floors result in higher claw growth with decreased claw angles, due to increased wear and tear.^5^

Cow gait is different between fore and hind limbs that result in different weight bearing pattern. In sound animals mechanical pressures produce a slight energy movement in the hooves that can help in hooves circulation.^6^ However, this pressure is higher in medial digit of the forelimb and lateral digit of the hind limb.^5^ Weight distribution between the two digits in forelimb is relatively equal but this distribution in hind limbs is mostly on the lateral claw.^7^ In an untrimmed cow 20% of the weight is on the medial claw of the hind limb and 80% is on the lateral one, this distribution changes to 30% and 70% on medial and lateral claw, respectively, after hoof trimming. This finding shows some imbalance even after hoof trimming.^8^

The main aim in Dutch-method of functional hoof trimming is achieving a flat weight bearing surface to provide maximum supportive area with reduction of pressure on the sole. This method increases contact area with a concomitant decrease of average claw pressure by ± 30% and also shifts some pressure from the lateral hind (± 10%) to the medial claw.^9^

It is recommended that cows be trimmed two times a year, heifers do not need trimming before parturition, unless showing apparent lameness or having very long toes (At least 10.00 to 12.00 cm).^10^ Effects of hoof trimming on the weight bearing patterns will stay for 26 weeks. In some farms this time may reduce to 4 months so in a herd with digital problems trimming should be done every 3 to 4 month (3 to 4 times a year).^11^^,^^12^

The changes in growth and wear rates result in changes in hoof conformation. The rate of growth of the hoof horn increase significantly after calving but the rate of wear does not increase. In contrast, the type of housing do not have significant effect on the growth of the hoof horn, but the heifers in straw yards have significantly lower rates of lateral horn wear.^13^

Long intervals of hoof trimming is known as the main reason for reduction of beneficial effects of hoof trimming in free stalls.^14^ More than a 7-month interval between two hoof trimmings result in more digital dermatitis, inter-digital dermatitis and heel erosion.^15^

However, Huber *et al*. reported an increase in heel erosion, digital dermatitis, laminitis and white line separation by decreasing hoof trimming intervals to less than four months.^16^ Hoof trimming on mid lactation reduced new lame cases by 25% at late lactation^17^ and before drying resulted in better hooves in parturition time.^18^

Shape of the hooves based on milking period and times to parturition, drying, and gestation could be different that may need more attention to this different shape in each trimming session. This study was done to evaluate common morphological findings of the lateral and medial hooves of forelimb in different hoof trimming timings in dairy cows.

## Materials and Methods

**Fig. 1:**
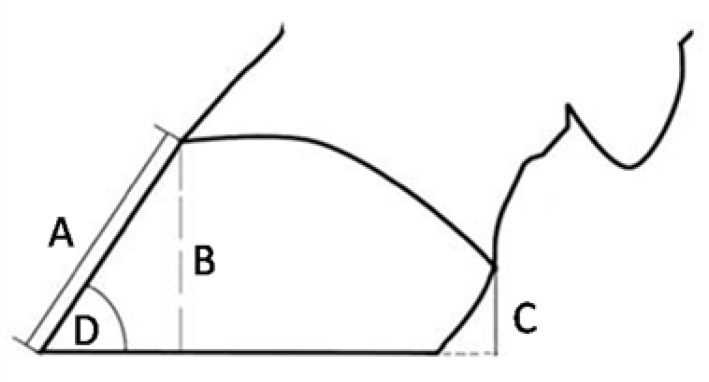
Hoof measurements; **A)** Toe length, **B)** Toe height, **C)** Heel height, **D)** Toe angle

This study was done in a dairy farm with 2200 milking cow, free stall barns, average daily production of 36 liters. Hoof care consisted regular hoof trimming in following times: 100-120 days in milk (DIM) (Group I), before drying (Group II), visual long toed cows (Group III) and delayed pregnant cows (Group IV). Forty cows from each group were selected randomly selected and following measurements were done after elevation of the hooves in trimming chute: toe length from coronary band to the toe tip (A), dorsal hoof angle ( ˚ ) measured between dorsal hoof wall and sole lines by a protractor (D), toe height (cm) from coronary band in toe region to the ground level (B), heel height (cm) from coronary band in heel region to the ground level (C) and heel height to toe length proportion (%), (Fig. 1).

**Data Analysis. **Data were analyzed by Student *t* test and one way analysis of variance (ANOVA), a Holm-Sidak test was used in case of any significance as a post hoc test in Sigmastat software (Jandel Scientific, San Rafael, USA). Group I was served as control group and all measurements were compared with this group. A *p* < 0.05 was considered as significant level.

## Results

Result of measurements in a total number of 160 cows in four different groups is reported in lateral and medial digits (Table 1). The highest toe length (9.19 ± 0.68) was recorded in medial digit of group IV and the lowest toe length (8.28 ± 0.62) was recorded in lateral digit of group I. Lateral digits in group IV and lateral digits of group III had the highest and the lowest toe height (7.29 ± 0.85 and 6.50 ± 0.76, respectively) as well as the lowest and the highest heel heights were recorded in medial digit of the group II (3.42 ± 0.75) and the lateral digits of the group IV (5.01 ± 1.02). The highest and the lowest toe angles were recorded in medial digits of the group IV (56.29 ± 7.14) and lateral digits of the group II (50.62 ± 7.93). Lateral digits in group IV (0.61 ± 0.13) and medial digits in group II (0.40 ± 0.08) got the highest and the lowest heel height to toe angle ratio, respectively.

**Table 1:** Mean ± SD of measurements in lateral and medial claws in experimental groups

**Groups**	**Measurements**					
	**Digit**	**Toe length (cm) ** *	**Toe height (cm)**	**Heel height (cm) ** *	**Toe angle ( ˚ ) **	**Heel height to toe length (%)** *
**I**	Lateral	8.28 ± 0.62	6.68 ± 0.83	4.06 ± 0.83	50.90 ± 7.01	0.52 ± 0.09
	Medial	8.88 ± 0.69	6.70 ± 0.48	3.63 ± 0.72	51.02 ± 4.66	0.43 ± 0.07
**II**	Lateral	8.50 ± 0.69	6.75 ± 0.61	4.00 ± 0.86	50.62 ± 7.93	0.50 ± 0.11
	Medial	8.98 ± 0.67	6.85 ± 0.57	3.42 ± 0.75	50.88 ± 8.05	0.40 ± 0.08
**III**	Lateral	8.58 ± 0.55	6.50 ± 0.76	4.29 ± 0.76	52.96 ± 5.03	0.53 ± 0.10
	Medial	8.93 ± 0.58	6.64 ± 0.77	3.85 ± 0.79	52.47 ± 7.11	0.45 ± 0.09
**IV**	Lateral	8.76 ± 0.73	7.29 ± 0.85	5.01 ± 1.02	56.14 ± 8.11	0.61 ± 0.13
	Medial	9.19 ± 0.68	7.02 ± 0.72	4.62 ± 0.82	56.29 ± 7.14	0.53 ± 0.09

* Asterisks indicate significant difference between lateral and medial digits (*p* < 0.05).

The toe length was recorded as 8.46, 9.22, 9.75 and 9.54 (cm) for group I, II, III and IV, respectively. Overall measurements in different groups under study are shown in Table 2. The highest measurements in all indices were recorded in group IV that was followed by group II except toe height that was higher in group II following group IV. The toe length was recorded significantly higher in all groups compared to those of group I, however, all other measurements in group IV were significantly higher than those of group I (Table 2).

**Table 2:** Mean ± SD of different measurements in both digits of experimental groups

**Measurements**	**Groups**			
	**I**	**II**	**III**	** IV**
**Toe length (cm)**	8.55 ± 0.71	8.74 ± 0.72 *	8.76 ± 0.59 *	8.98 ± 0.74 *
**Toe height (cm)**	6.69 ± 0.68	6.80 ± 0.59	6.61 ± 0.76	7.15 ± 0.79 *
**Heel height (cm)**	3.84 ± 0.81	3.73 ± 0.84	4.07 ± 0.80 *	4.81 ± 0.95 *
**Angle degree ( ˚ )**	50.98 ± 5.93	50.75 ± 7.97	52.71 ± 6.15	56.14 ± 7.60 *
**Heel height to toe length (%)**	0.47 ± 0.09	0.45 ± 0.11	0.49 ± 0.10	0.57 ± 0.12*

* Asterisks indicate significant difference recorded with group I (*p* < 0.05).

## Discussion

Hoof trimming in group I was done after a period of metabolic stress and negative energy balance. Group II cows were trimmed based on pregnancy and in case of any reproduction problem, time distance between these two hoof trimmings was extended that resulted in over-growing of the claws. Such a cow (Group IV) was detected by a formula based on average open days of the herd.^19^ Visual detection of the long toed cows is the main, producing cows of the herd annually.^20^ However Van der Tol* et al*. recommended 2 to 3 times of hoof trimming in a year,^8^ Manske *et al*. reported reasonable results by two times of hoof trimming in a year, it was reported that lameness and other problems related to laminitis (specially sole ulcer) could be reduced by a hoof trimming 4-5 months before the high risk season.^21^ Dorsal hoof wall in toe region by its most tubular density is the hardest part of the hoof that result in rapid growth.^22^ Group III cows showed longer toes (over 8.00 cm), showing toe length that was considered a factor in detecting such a cows. 

Based on average of 130 days open in this herd, around 230 days took between hoof trimming of the cows between groups I and II and during this time just 0.19 cm growth recorded based on the average toe length. However, cow’s hoof normally grow as 0.50 cm per month, showing that wearing also is a very important finding among these cows that may result in thin sole.^23^

Hoof overgrowth results in imbalance of the digits and weight bearing pattern changes that is not the case in long toes visually detected in cows. However, Nuss and Paulus reported the effect of age on longer toes,^24^ and size of the animals also has been proven to affect toe length.^25^ Difference between two digits toe length is considered as factor that can show demand of hoof trimming, in this study all groups showed significant difference in toe length and medial claw had longer toes than lateral ones, that is the case of previous reports.^26^^,^^27^ This difference shows that the animal needs trimming and making toes as equal as possible. The lowest toe length is recorded in group I that needs more attention due to the danger of over trimming and its complications in this group.^23^

Although number of cows with 8.00 cm and higher toe length are higher in group III, the average of the toe length is slightly higher in group IV than group III. Using previously mentioned formula for detecting delay pregnancy cows could help find long toe cows as well as visual detection of such cows. 

Increasing toe height, toe angle and heel height in group IV show less wearing in all parts or may be a result in more wearing in toe region. These cows are prone to thin sole in toe region,^23^ and consequently toe ulcers, so diagnosing and treating these cows play a very important role in the herd. However, the lowest height was recorded in group III and the highest one was recorded in group IV. This index is reported by Somer which was 6.71 cm that is close to our findings.^28^

Following toe growth heel height should normally decrease,^29^ and weight shifts toward the heel.^30^ In this study toe length is longer in group IV than other groups as well as heel height that was higher than other groups. Two types of overgrowth is defined, the first one is balanced grow (toe and heel height increase in a similar rate, that is the case in cows on soft beddings) and the second one is free stall type of hoof growth.^10^ Results of this study showed the first type of hoof growth in group IV. However, this finding regarding freestall barn usage in all groups need more investigation. An important relation between heel height and toe length resulted in a balance between cranial and caudal part of a digit.^10^ Higher ratios show a balance growth of the hoof and equal growth and wear of the heel and the toe. This ratio was higher in group IV than other groups that can be a result of balance growth of the feet and more wear in toe region.

Imbalance between heel heights of two digits may result in injuries like sole and heel ulcers.^26^ Heel height was different in all groups under study, that indicate the necessity of hoof trimming in all groups. Heel height was reported as 3.00 to 4.00 cm,^25^ that can change from 2.50 to 3.50 in younger and 3.00 to 4.50 cm in older cows^31^ that is the case in present study. However, Winkler and Margerison reported 5.20 to 6.30 cm of heel height in cow in Holstein breed.^32^

Hoof trimming resulted in higher toe angle that help in more stability of the hooves and increasing heel height and finally reducing conditions like digital dermatitis and foot rot.^33^ Toe angle can increase by growth of heel bulb in zone 6 of the hooves and also wearing of the toe,^28^^,^^34^ on other hand increasing toe length in overgrown hooves results in decreasing toe angle.^22^ This angle is reported as 50 degree in forelimbs.^25^^,^^35^^,^^36^ Blowey reported 45 to 50 degree for the best weight bearing in hooves.^31^ However, Burgi and Cook believe that toe angles of more than 52 results in more protection of the digital cushions and postpone claw horn defects.^33^ In current study highest toe angle was recorded in group IV and the least one in group II, however, all measurements were above 50 degree.

## References

[1] Hubbert WT (1974). Relationship of unkeratinized skin to bovine fetal mummification: A hypothesis. Can J Comp Med.

[2] Collick DW, Ward WR, Dobson H (1989). Associations between types of lameness and fertility. Vet Rec.

[3] Fourichon C, Seegers H, Bareille N (1999). Effects of disease on milk production in the dairy cow: A review. Prev Vet Med.

[4] Haskell MJ, Rennie LJ, Bowell VA (2006). Housing system, milk production, and zero-grazing effects on lameness and leg injury in dairy cows. J Dairy Sci.

[5] Van der Tol PPJ, Metz JHM, Noordhuizen-Stassen EN (2002). The pressure distribution under the bovine claw during square standing on a flat substrate. J Dairy Sci.

[6] Mülling CKW, Greenough PR (2006). Functional synergism of the biomechanical systems of the bovine claw.

[7] Van der Tol PPJ, Metz JHM, Noordhuizen-Stassen N (2003). The vertical ground reaction force and the pressure distribution on the claws of dairy cows while walking on a flat substrate. J Dairy Sci.

[8] Van der Tol PPJ, Metz JHM, Noordhuizen-Stassen EN (2004). The force and pressure distribution on the claws of cattle and the biomechanical effect of preventive trimming.

[9] Toussaint-Raven E (1973). Determination of weight bearing by the cows foot. Dutch J Vet Med.

[10] Greenough PR (2007). Bovine laminitis and lameness: A hands-on approach.

[11] Kehler W, Gerwing T (2004). Effects of functional claw trimming on pressure distribution under hind claws of German Holstein cows.

[12] Meyer SW, Weishaupt MA, Nuss KA (2007). Gait pattern of heifers before and after claw trimming: A high-speed cinematographic study on a treadmill. J Dairy Sci.

[13] Livesey CT, Laven RA (2007). Effects of housing and intake of methionine on the growth and wear of hoof horn and the conformation of the hooves of first-lactation Holstein heifers. Vet Rec.

[14] Fjeldaas T, Sogstad AM, Østerås O (2006). Claw trimming routines in relation to claw lesions, claw shape and lameness in Norwegian dairy herds housed in tie stalls and free stalls. Prev Vet Med.

[15] Somers JGCJ (2004). Claw disorders and disturbed locomotion in dairy cows: The effect of floor systems and implications for animal welfare [PhD Thesis].

[16] Huang YC, Shanks RD, McCoy GC (1995). Evaluation of fixed factors affecting hoof health. Livest Prod Sci.

[17] Hernandez JA, Garbarino EJ, Shearer JK (2006). Prophylactic efficacy of hoof health examination and hoof trimming during mid-lactation to reduce incidence of lameness in Holstein cows during late lactation.

[18] Andrews AH, Blowey RW, Boyd H (2004). Bovine medicine: Diseases and husbandry of cattle.

[19] Mohammadnia AR, Khaghani A, Ghorbani Z (2011). A new cut point for bovine hoof trimming.

[20] Mohammadnia AR, Gholami M, Khaghani A (2010). Necessity and the manner of implementation of the hoof trimming programs in dairy farms.

[21] Manske T, Hultgren J, Bergsten C (2002). The effect of claw trimming on the hoof health of Swedish dairy cattle. Prev Vet Med.

[22] Blowey RW, Shearer JK (2002). Claw trimming-How should it be done? A comparison of two approaches.

[23] Van Amstel SR, Shearer JK, Palin FL (2004). Moisture content, thickness, and lesions of sole horn associated with thin soles in dairy cattle. J Dairy Sci.

[24] Nuss K, Paulus N (2006). Measurements of claw dimensions in cows before and after functional trimming: A post-mortem study. Vet J.

[25] Weaver AD, Jean GS, Steiner A (2005). Bovine surgery and lameness.

[26] Anderson DE, Rings DM (2009). Current veterinary therapy: Food animal practice.

[27] Mgasa MN, Kempson SA (2002). Functional anatomy of the laminar region of normal bovine claws.

[28] Somers JGCJ, Schouten WGP, Frankena K (2005). Development of claw traits and claw lesions in dairy cows kept on different floor systems. J Dairy Sci.

[29] Shearer JK, Van Amstel SR (2007). Effect of flooring and/or flooring surfaces on lameness disorders in dairy cattle.

[30] Raven ET (1989). Cattle foot care and claw trimming.

[31] Blowey RW (1998). Cattle lameness and hoof care.

[32] Winkler B, Margerison JK (2005). Hoof measurements and their relationship to lameness in first lactation heifers.

[33] Burgi K, Cook N (2008). Three adaptions to the functional trimming method.

[34] Greenough PR, Vermunt JJ (1991). Evaluation of subclinical laminitis in a dairy herd and observations on associated nutritional and management factors. Vet Rec.

[35] Fessl L (1982). On standardization of claw measurements in cattle.

[36] McDaniel BT, Verbeek B, Wilk JC (1984). Relationships between hoof measures, stayabilities, reproduction and changes in milk yield from first to later lactations. J Dairy Sci.

